# Influence of corneal power on intraocular lens power of the second eye in the SRK/T formula in bilateral cataract surgery

**DOI:** 10.1186/s12886-017-0664-3

**Published:** 2017-12-28

**Authors:** Young Choi, Youngsub Eom, Jong Suk Song, Hyo Myung Kim

**Affiliations:** 1 Department of Ophthalmology, Korea University College of Medicine, Seoul, South Korea; 2 Department of Ophthalmology, Ansan Hospital, Korea University College of Medicine, 123, Jeokgeum-ro, Danwon-gu, Ansan-si, Gyeonggi-do 15355 South Korea

**Keywords:** Bilateral cataract extraction, Corneal power, Intraocular lens power, SRK/T formula

## Abstract

**Background:**

To evaluate the effect of different adjustments of the refractive outcome of the first eye according to corneal power (K) in order to improve the intraocular lens (IOL) power calculation of the second eye in the SRK/T formula.

**Methods:**

One hundred thirty-four patients who underwent uncomplicated bilateral, sequential phacoemulsification with AcrySof IQ implantation were enrolled. The optimal partial adjustment of the refractive outcome of the first eye according to K was retrospectively analyzed using a regression formula.

**Results:**

In all patients, the optimal partial adjustment of the refractive outcome of the first eye was calculated as 56%. For K values between 42.8 D and 44.6 D, the optimal partial adjustment was calculated as 30%; however, this adjustment of the first eye did not significantly improve the refractive outcome in the second eye of the subgroup with K values between 42.8 D and 44.6 D. For K values greater than 44.6 D or less than 42.8 D, the optimal partial adjustments were calculated as 69% and 81%, respectively. According to these results, the adjustment of the first eye significantly improved the refractive outcome in the second eye from 0.36 to 0.26 D (*P* < 0.001) in the entire data set. This result was significantly lower than that using a single partial adjustment (56%) (0.28 D; *P* = 0.027).

**Conclusions:**

For K values greater than 44.6 D or less than 42.8 D, an approximately 70–80% adjustment of the first eye error should be considered. In contrast, for K values between 42.8 D and 44.6 D, a 30% or less adjustment should be considered in the SRK/T formula.

## Background

Postoperative vision after cataract surgery has been greatly improved by advances in surgical techniques, precise biometry techniques, and intraocular lens (IOL) power calculation formulas [1–7]; however, the refractive error is still a major concern in cataract surgery.

A previous study demonstrated that using corneal power (K)-specific constants improved the refractive outcomes predicted by the Sanders-Retzlaff-Kraff (SRK)/T formula because it predicts a myopic refractive error for a steep cornea and a hyperopic refractive error for a flat cornea [4]. In that study, the refractive error showed a distribution between 0.25 D to −1.00 D for K values between 46.0 and 47.0 D. In cases of bilateral, sequential cataract surgery, previous studies have shown that the refractive outcome of the first eye can be used to improve the IOL calculation for the second eye due to the symmetry between the two eyes [8–10]. Thus, if the first eye shows a − 1.00 D myopic shift with a K of 47 D, the second eye is likely to show a similar myopic shift, which is different from the average refractive error. Therefore, we hypothesized that increasing the magnitude of the adjustment for the first eye error for a steep or flat cornea would improve the refractive outcome in the second eye when using the SRK/T formula. This study was designed to evaluate the effect of different adjustments of the refractive outcome of the first eye according to K for improving the IOL power calculation of the second eye in the SRK/T formula.

## Methods

### Study population

This retrospective cross-sectional study included 268 eyes from 134 patients who underwent uncomplicated bilateral, sequential phacoemulsification with IOL implantation at Korea University Anam Hospital, Seoul, Korea between April 2008 and December 2015. An AcrySof IQ (SN60WF, Alcon, Fort Worth, TX, USA) IOL was implanted in both eyes of each patient. Patients who had best corrected visual acuities (BCVA) better than or equal to 20/40 in both eyes after cataract surgery were included. Patients with a traumatic cataract, prior ocular surgery (such as penetrating keratoplasty or refractive surgery), complicated surgery (such as posterior capsule rupture), or postoperative complications were excluded. Institutional Review Board (IRB) approval was obtained from Korea University Anam Hospital, Seoul, Korea for this study. All research and data collection methods followed the tenets of the Helsinki agreement. The data used in this study were de-identified for the sake of privacy for subjects.

### Patient examination

All measurements were taken by a trained ophthalmic examiner who measured the preoperative axial length (AL) and K with optical biometry using an IOLMaster version 5.02 or higher (Carl Zeiss Meditech, Jena, Germany). IOL power was calculated using the SRK/T formula of the IOLMaster. The data-adjusted A-constant for AcrySof IQ was 119.0, calculated in our previous study using the Haigis constant optimization Excel spreadsheet (Microsoft Inc., Redmond, WA, USA) for optical biometry [4, 11].

Postoperative uncorrected visual acuity (UCVA), manifest refraction, and BCVA were measured at postoperative visits between three and 10 weeks.

### Surgical technique

Phacoemulsification and IOL implantation were performed under topical anesthesia with 0.5% proparacaine hydrochloride (Alcaine; Alcon, Fort Worth, TX, USA) via a 2.2 or 2.75 mm temporal clear corneal incision by an experienced surgeon (HM.K.). The IOL was inserted into the capsular bag.

### Main outcome measure(s)

The refractive error was defined as the difference between the observed refractive spherical equivalent three to 10 weeks postoperatively and the predicted refraction (spherical equivalent) by IOLMaster using the SRK/T formula (refractive error = postoperative spherical equivalent – preoperative predicted refraction). The mean absolute refractive error (MAE) was defined as the mean absolute value of the refractive error.

The optimal partial adjustment of the refractive error of the first eye according to K for improving the IOL calculation of the second eye was analyzed in retrospect using the corrective regression formula [9]: *R*ϰ_cor_ = *R*ϰ_exp_ + β x *P*ϰ_err_, where *R*ϰ_cor_ is the observed refractive spherical equivalent of the second eye, *R*ϰ_exp_ is the expected refractive prediction of the second eye, *P*ϰ_err_ is the refractive error of the first eye, and β is a correlation coefficient that is a magnitude of adjustment of first-eye error for improving the refractive outcome of the second eye.

The adjusted MAE of the second eye without considering K (MAE_WCP_) was defined as the MAE of the second eye using a partial adjustment for the refractive error of the first eye with a calculated correlation coefficient from the entire data set by the corrective regression formula. The adjusted MAE of the second eye according to K (MAE_ACP_) was defined as the MAE of the second eye using a partial adjustment for the refractive error of the first eye with calculated correlation coefficients for within cut-off and outside cut-off values. To decide the K cut-off value, the correlation coefficient for the partial adjustment was calculated from the cumulative subgroups based on K. Each cumulative subgroup was made according to both an increase in K from 42 to 47 D and a decrease in K from 47 to 42 D at 0.2 D intervals. After that, the lower and upper cut-off K values, which showed a deviation from the correlation coefficient increasing or decreasing trend, were decided.

### Statistical analysis

Descriptive statistics were obtained using the Statistical Package for the Social Sciences (SPSS) version 21.0 (IBM Corp., Armonk, NY, USA). The Kolmogorov-Smirnov test was performed to assess data distribution normality. Paired *t*-tests were used for parametric continuous variables and the Wilcoxon signed rank test was used for nonparametric continuous variables according to the results of normality distribution tests. Repeated-measures analysis of variance (ANOVA) with the Bonferroni correction were performed to assess statistical differences among the unadjusted MAE, MAE_WCP_, and MAE_ACP_. Results were considered statistically significant if the *p*-value was less than 0.05. A post-hoc power analysis using the Wilcoxon signed-rank test option of G*power, version 3.1.9.2 (Franz Paul, Kiel, Germany), was conducted to determine study power.

## Results

One hundred thirty-four patients were included in this study. Of the 134 patients, 50 (37.3%) were men and 84 were women. The mean age (± SD) was 68.6 ± 8.5 years (range, 43 to 90 years). The mean K, AL, calculated IOL power, preoperative predicted refraction, postoperative refraction, and refractive error are shown in Table 1.

**Table 1:** Clinical characteristics of patients and eyes included in the present study (*n* = 134)

Parameter	Patients	First eye	Second eye	*P* value^a^
Age, years (SD)	68.6 (8.5)			–
Sex (Male:Female) (%)	50 (37.3): 84 (62.7)			–
Corneal power, D (SD)		44.22 (1.43)	44.20 (1.47)	0.687
Axial length, mm (SD)		23.55 (0.95)	23.52 (0.89)	0.691^b^
IOL power, D (SD)		20.8 (2.7)	20.9 (2.4)	0.395^b^
Predicted refraction, D (SD)		−0.26 (0.24)	−0.26 (0.23)	0.847
Refraction at postop 3 to 10 weeks, D (SD)		−0.22 (0.55)	−0.21 (0.50)	0.884^b^
Refractive error, D (SD)		0.04 (0.49)	0.04 (0.45)	0.990

Data are mean (SD) except for parameter sex, which are *n* (%)

*SD* standard deviation, *D* diopters, *OL* intraocular lens

^a^Paired *t*-test was used for parametric continuous variables

^b^Wilcoxon signed rank test was used for nonparametric continuous variables

**Fig. 1:**
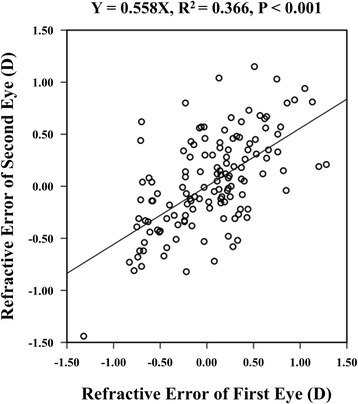
Interocular correlation of the refractive error with the Sanders-Retzlaff-Kraff (SRK)/T formula. D = diopters

There was a significant correlation in K (R^2^ = 0.915, *P* < 0.001), AL (R^2^ = 0.912, P < 0.001), and IOL power (R^2^ = 0.883, P < 0.001) between the first and second eyes. There was also a significant correlation between the refractive error of the first and second eyes (R^2^ = 0.366, P < 0.001; Fig. 1). According to the corrective regression formula^6^, the correlation coefficient (β) was 0.56 in all patients using the SRK/T formula. This means that the optimal partial adjustment of the refractive error of the first eye for IOL calculation of the second eye was determined to be 56%. According to these results, the MAE of second eyes decreased from 0.36 to 0.28 D (*P* < 0.001; Table 2).

**Table 2:** Comparison of the unadjusted mean absolute refractive error (MAE_UNADJ_), adjusted MAE without considering corneal power (MAE_WCP_), and adjusted MAE according to corneal power (MAE_ACP_) of the second eye in each subgroup (Repeated measures ANOVA with Bonferroni correction)

MAE of second eye (D)		Total (*n* = 134)	Within cut-off values [line break] 42.8D ≤ K < 44.6D (*n* = 64)	Outside cut-off values [line break] K < 42.8D or 44.6D ≤ K (*n* = 70)
Unadjusted MAE (MAE_UNADJ_), D (SD)		0.36 (0.27)	0.33 (0.26)	0.40 (0.28)
Adjusted MAE without considering corneal power (MAE_WCP_), D (SD)		0.28 (0.22)	0.33 (0.24)	0.24 (0.20)
Adjusted MAE according to corneal power (MAE_ACP_), D (SD)		0.26 (0.23)	0.31 (0.24)	0.21 (0.21)
*P* value	MAE_UNADJ_ vs. MAE_WCP_	< 0.001	> 0.999	< 0.001
	MAE_UNADJ_ vs. MAE_ACP_	< 0.001	> 0.999	< 0.001
	MAE_WCP_ vs. MAE_ACP_	0.027	0.549	0.032

*D* diopters, *MAE* mean absolute refractive error, *K* mean corneal power, *SD* standard deviation

**Fig. 2:**
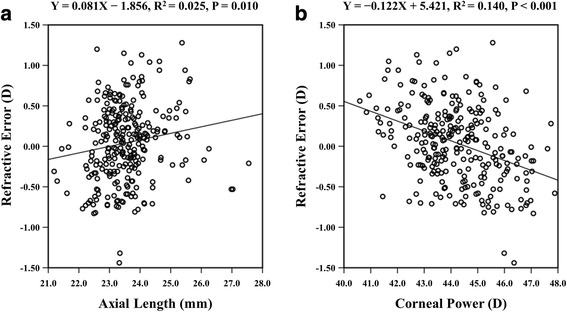
Comparison of axial length, corneal power, and refractive error with the Sanders-Retzlaff-Kraff (SRK)/T formula in both eyes (*n* = 268). **a** Relation between axial length and refractive error. **b** Relation between corneal power and refractive error. D = diopters

There was a very weak positive correlation between AL and refractive error (R^2^ = 0.025, *P* = 0.010; Fig. 2a). On the other hand, a negative correlation was observed between K and refractive error (R^2^ = 0.140, P < 0.001; Fig. 2b). According to the regression equation, the refractive error could be zero when K was 44.43 D. As K increased, the refractive error showed a tendency for myopic refractive outcomes. On the contrary, as K decreased, the refractive error showed a tendency for hyperopic refractive outcomes.

**Fig. 3:**
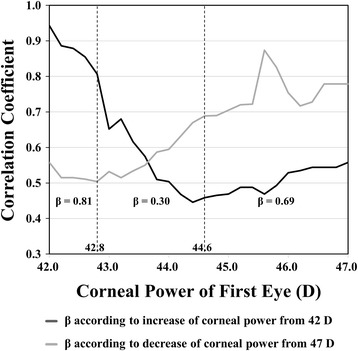
Graph showing the correlation coefficient according to corneal power. The correlation coefficients (Y-axis) of each cumulative subgroup (X-axis), which contained subjects whose corneal power was less than the corneal power on the X-axis, are shown as a graph according to the increase of corneal power from 42 D (heavy line). The correlation coefficient of each cumulative subgroup, which contained subjects whose corneal power was greater than or equal to the corneal power on the X-axis, are shown as a graph according to the decrease of corneal power from 47 D (light line). β = Correlation coefficient

Figure 3 shows the correlation coefficients of each cumulative subgroup which were calculated according to both an increase in K from 42 D (heavy line) and a decrease in K from 47 D (light line). The correlation coefficients of the cumulative subgroups tended to decrease as K increased from 42D and tended to decrease as K decreased from 47 D. The lower and upper cut-off K values were determined to be 42.8 and 44.6 D, respectively. The correlation coefficient for values less than the lower cut-off (K < 42.8 D) was calculated as 0.81 and that for values over the upper cut-off (44.6 D ≤ K) was calculated as 0.69. The lowest correlation coefficient was observed between 42.8 and 44.6 D (correlation coefficient = 0.30).

**Fig. 4:**
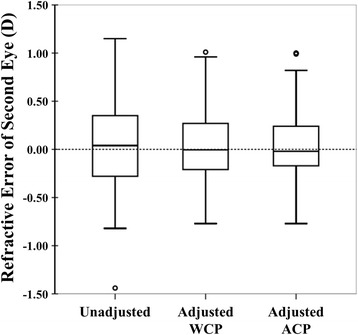
The refractive error of the second eye was calculated using unadjustment, partial adjustment without considering corneal power (WCP), and partial adjustment according to corneal power (ACP) of the refractive error of the first eye using the Sanders-Retzlaff-Kraff (SRK)/T formula. D = diopters

The MAE_ACP_ (± SD) (0.26 ± 0.23 D) was smaller than the MAE_WCP_ (0.28 ± 0.22 D) in the entire dataset (*P* = 0.027; Table 2 and Fig. 4). In a subgroup analysis, neither the MAE_WCP_ nor MAE_ACP_ of the subgroup within the cut-off values improved the refractive outcome in the second eye. Otherwise, both the MAE_WCP_ and MAE_ACP_ of the subgroup outside the cut-off values significantly improved the refractive outcome from 0.40 D to 0.24 D and 0.21 D, respectively (*P* < 0.001, P < 0.001, respectively). The MAE_ACP_ (0.21 ± 0.21 D) was significantly smaller than the MAE_WCP_ (0.24 ± 0.20 D) in the subgroup outside of the cut-off values (*P* = 0.032).

The MAE_ACP_ and MAE_WCP_ in the subgroup outside of the cut-off values were used in a post-hoc power analysis. The correlation between the MAE_ACP_ and MAE_WCP_ was 0.925 and the effect size was 0.375. The effect size of 0.375 and a two-tailed alpha of 0.05 in the subgroup analysis with 70 patients led to a power of 0.86.

## Discussion

A high degree of interocular symmetry of biometry between the two eyes is helpful in IOL power calculation for the second eye in bilateral sequential cataract surgery [12]. Most patients in this study showed strong interocular correlation with K (R^2^ = 0.915), AL (R^2^ = 0.912), and IOL power (R^2^ = 0.883). Similarly, Covert et al., [8] showed strong interocular correlations with K (R^2^ = 0.88) and AL measurements (R^2^ = 0.96) and several other studies have shown a high degree of interocular correlation with K, anterior chamber depth, and AL measurements [10, 12, 13]. Actually, refractive outcome of the second eye was improved using the refractive error observed in the first eye [8–10].

This study evaluated the effect of different adjustments of the refractive error observed in the first eye according to K on the refractive outcome of the second eye using the SRK/T formula. The results showed that the method of different adjustments according to K value significantly improved the refractive outcome of the second eye compared to the method of fixed partial adjustments. When corneal power was not considered, the optimal partial adjustment of the refractive error of the first eye was calculated to be 56% in the entire dataset. The results of the present study using the SRK/T formula were similar to the previous studies. Covert et al. [8] performed a study demonstrating the effectiveness of a partial adjustment (50%) to the refractive error observed in the first eye for IOL power calculation of the second eye to improve the refractive outcome of the second eye using the Holladay I and SRK II formulas. Olsen [9] demonstrated similar results using the SRK II (56%), SRK/T (38%), and more recent Olsen formulas (27%). Otherwise, there was no benefit to full adjustment of the refractive error of the first eye [8, 13].

Olsen [9] showed that the magnitude of the partial adjustment of refractive outcome of the first eye and the improvement in refractive outcome of the second eye differ depending on the IOL power calculation formula. When the formula was less accurate, more adjustments were needed, and greater benefits were shown after the correction. In the present study, the correlation coefficients of each cumulative subgroup were calculated to determine the cut-off values according to K and the cut-off values were set at 42.8 D and 44.6D. When the cornea was steeper than the upper cut-off value or flatter than the lower cut-off value, the magnitude of optimal partial adjustment of the first eye refractive error (69%, 81%, respectively) were larger than that of the whole period (56%). The lowest magnitude of optimal partial adjustment (30%) was observed within the cut-off values. There was no benefit to partial adjustment for first eye error within the cut-off values. Otherwise, there was an improvement in the refractive outcome in the subgroups outside of the cut-off values. The MAE_ACP_ showed a greater effect than the MAE_WCP_ in the subgroup outside of the cut-off values. Thus, the magnitude of the partial adjustment of the first eye refractive error and the improvement in refractive outcome of the second eye could differ depending on the K in the SRK/T formula.

Sheard et al., [14] demonstrated that the SRK/T formula has non-physiologic behavior in the corrected AL and corneal height calculation. According to the non-physiologic behavior in the corneal height calculation, [14] the predicted corneal height tends to be overestimated as K increases and tends to be underestimated as K decreases in the SRK/T formula. Our previous study [4] and the present study demonstrated a negative correlation between K and the refractive error using the SRK/T formula. In the present study, the refractive error was smallest when K was 44.43 D. The refractive outcome became more myopic as K increased and became more hyperopic as K decreased. These findings were similar to those noted above [14]. These results imply that the accuracy of the SRK/T formula decreases when the cornea becomes steeper or flatter. Therefore, the magnitude of adjustment of the first eye outcome should be changed according to K in the SRK/T formula.

There are some limitations in the present study. First, the sample size was relatively small and medical records were retrospectively reviewed. Second, UCVA, manifest refraction, and BCVA were measured at postoperative visits between three and 10 weeks, due to the retrospective nature of this study [6]. However, a previous study showed that the changes in effective lens position and refractive error of the in-the-bag AcrySof IOL were insignificant from 1 week to 6 months after surgery [15]. Third, there were a few patients who had severe differences in K between both eyes, and the differences in K were not considered in the present study. Therefore, a study on the optimal partial adjustment of the refractive error of the first eye according to K with a large number of patients will be necessary.

## Conclusions

Partial adjustment of the refractive error of the first eye according to the regression formula improved the refractive outcome in the second eye. When the cornea is steep or flat, an approximately 70–80% magnitude adjustment of the first eye error should be considered in the SRK/T formula. On the contrary, when K is within the range of cut-off values, a magnitude adjustment of 30% or less should be considered.

## Abbreviations

AL: Axial length
BCVA: Best corrected visual acuity
IOL: Intraocular lens
K: Corneal power
MAE: Mean absolute refractive error
MAE_ACP_: Adjusted MAE of the second eye according to K
MAE_WCP_: Adjusted MAE of the second eye without considering K
SRK/T: Sanders-Retzlaff-Kraff /T
UCVA: Uncorrected visual acuity
β: Correlation coefficient
